# Imaging atherosclerotic plaques by targeting Galectin-3 and activated macrophages using (^89^Zr)-DFO- Galectin3-F(ab')_2_ mAb

**DOI:** 10.7150/thno.50247

**Published:** 2021-01-01

**Authors:** Zohreh Varasteh, Francesco De Rose, Sarajo Mohanta, Yuanfang Li, Xi Zhang, Benedikt Miritsch, Giorgia Scafetta, Changjun Yin, Hendrik B. Sager, Sarah Glasl, Dimitris Gorpas, Andreas J.R. Habenicht, Vasilis Ntziachristos, Wolfgang A. Weber, Armando Bartolazzi, Markus Schwaiger, Calogero D'Alessandria

**Affiliations:** 1 Department of Nuclear Medicine, Klinikum rechts der Isar der TUM, Munich, Germany.; 2 Institute for Cardiovascular Prevention, University Hospital of Ludwig-Maximilians-University, Munich, Germany.; 3 Department of Cardiology, German Heart Center Munich, Munich, Germany.; 4 Deutsches Zentrum für Herz-Kreislauf-Forschung (DZHK) e.V., partner site Munich Heart Alliance.; 5 Institute of Biological and Medical Imaging, Helmholtz Zentrum München, Munich, Germany.; 6 Chair of Biological Imaging and Center for Transnational Cancer Research (TranslaTUM), Technical University of Munich, Munich, Germany.; 7 Pathology Research Laboratory, Cancer Center Karolinska, SE-17176 Stockholm, Sweden.; 8 Pathology Research Laboratory, St. Andrea University Hospital, Rome, Italy.

**Keywords:** Atherosclerotic plaques, Inflammation, activated macrophages, PET/CT, Galectin-3

## Abstract

**Rationale:** The high expression of Galectin-3 (Gal3) in macrophages of atherosclerotic plaques suggests its participation in atherosclerosis pathogenesis, and raises the possibility to use it as a target to image disease severity *in vivo*. Here, we explored the feasibility of tracking atherosclerosis by targeting Gal3 expression in plaques of apolipoprotein E knockout (ApoE-KO) mice *via* PET imaging.

**Methods:** Targeting of Gal3 in M0-, M1- and M2 (M2a/M2c)-polarized macrophages was assessed *in vitro* using a Gal3-F(ab')_2_ mAb labeled with AlexaFluor®488 and ^89^Zr- desferrioxamine-thioureyl-phenyl-isothiocyanate (DFO). To visualize plaques *in vivo*, ApoE-KO mice were injected i.v. with ^89^Zr-DFO-Gal3-F(ab')_2_ mAb and imaged via PET/CT 48 h post injection. Whole length aortas harvested from euthanized mice were processed for Sudan-IV staining, autoradiography, and immunostaining for Gal3, CD68 and α-SMA expression. To confirm accumulation of the tracer in plaques, ApoE-KO mice were injected i.v. with Cy5.5-Gal3-F(ab')_2_ mAb, euthanized 48 h post injection, followed by cryosections of the body and acquisition of fluorescent images. To explore the clinical potential of this imaging modality, immunostaining for Gal3, CD68 and α-SMA expression were carried out in human plaques. Single cell RNA sequencing (scRNA-Seq) analyses were performed to measure LGALS3 (i.e. a synonym for Gal3) gene expression in each macrophage of several subtypes present in murine or human plaques.

**Results:** Preferential binding to M2 macrophages was observed with both AlexaFluor®488-Gal3-F(ab')_2_ and ^89^Zr-DFO-Gal3-F(ab')_2_ mAbs. Focal and specific ^89^Zr-DFO-Gal3-F(ab')_2_ mAb uptake was detected in plaques of ApoE-KO mice by PET/CT. Autoradiography and immunohistochemical analyses of aortas confirmed the expression of Gal3 within plaques mainly in macrophages. Moreover, a specific fluorescent signal was visualized within the lesions of vascular structures burdened by plaques in mice. Gal3 expression in human plaques showed similar Gal3 expression patterns when compared to their murine counterparts.

**Conclusions:** Our data reveal that ^89^Zr-DFO-Gal3-F(ab')_2_ mAb PET/CT is a potentially novel tool to image atherosclerotic plaques at different stages of development, allowing knowledge-based tailored individual intervention in clinically significant disease.

## Introduction

Atherosclerosis is a multifactorial chronic inflammatory disorder characterized by a disturbed equilibrium of immune responses and lipid accumulation, leading to plaque development 1. It is an age-dependent disease and the most common cause of death worldwide 2. Most atherosclerotic plaques remain clinically asymptomatic, though some undergo a series of changes that cause life-threatening complications 3. These changes include patches of calcifications, ulcerations associated with shedding of cholesterol emboli into the bloodstream, superimposed thrombosis in areas of plaque fissures, haemorrhage, and aneurysmal dilatations. These changes make plaques vulnerable and prone to events such as myocardial infarction and stroke 4.

Visualization of atherosclerotic plaques and in particular identification of the high-risk lesions prone to rupture *in vivo* is a challenging objective with the ultimate goal to develop pharmacological and/or therapeutic strategies to prevent lethality 5. Though conventional diagnostic tools yield anatomical and morphological information, they are still of limited value to identify vulnerable plaques and to predict their risk to rupture and subsequent complications 6. Therefore, to detect vulnerability *in vivo*, imaging approaches are highly relevant to facilitate decisions by the medical community when and how to apply invasive therapies to prevent clinical sequelae of the disease. Molecular imaging offers potential opportunities to develop diagnostic approaches to assess the pathobiology of atherosclerotic plaques. Improved understanding of the molecular and biological processes has stimulated the development of imaging probes, which may aid to identify high-risk atherosclerotic lesions and to apply individually tailored interventions 6. Since atherosclerosis is a systemic arterial wall disease, molecular imaging of plaques should preferably be performed by whole-body imaging technologies, such as positron emission tomography (PET) imaging.

Monocyte-derived macrophages are major inflammatory cells associated with atherosclerotic lesions and are recognized as key pathophysiologically important immune cells. Therefore, macrophages are gaining attention as imaging targets in atherosclerosis 7-10.

Galectin-3 (Gal3) is a member of the lectin family, which is known to be involved in multiple aspects of inflammatory cell pathology 11. It is a constitutive marker of activated macrophages 12,13. Overexpression of Gal3 in the aorta of hypercholesterolemic animals and in human atherosclerotic lesions has been reported, suggesting a direct involvement of this multifaceted protein in the pathophysiology of the disease 14-16. Gal3 has therefore been proposed as a potential biomarker for culprit lesions 17. These characteristics make Gal3 a potential target for non-invasive molecular imaging of atherosclerosis and in particular plaque vulnerability. Radiotracers based on a full Gal3 monoclonal antibody (mAb) and its F(ab')_2_ fragment derivative have been developed and used for non-invasive imaging of Gal3-expressing tumors 18-20. Here, we aimed at investigating whether it is feasible to specifically target and visualize Gal3 expression on the cells present in plaques, *i.e.* subset of macrophages and to a lesser extent other Gal3 positive cell types, using a Gal3-specific probe in apolipoprotein E-knockout (ApoE-KO) mice.

Using zirconium-89 labeled Gal3-F(ab')_2_ mAb, we show that nuclear imaging has the potential to visualize specific biological activities associated with plaque progression and/or instability, and that Gal3 expression in atherosclerotic plaques may be used as a novel biomarker to identify patients at high risk of cardiovascular events or to monitor the impact of treatment aimed at plaque stabilization. Using fluorophore coupled Gal3-F(ab')_2_ mAb, we delineated the distribution of the probe within the plaques with high resolution, providing initial evidence of the potential sensitivity of the proposed imaging approach.

## Methods

### Animals

Adult ApoE-KO mice (female, 31-week-old, 29-34 g weight, from Jackson laboratory on the C57BL/6 genetic background) were used for *in vivo* and *ex vivo* imaging studies. Mice were on high fat Western diet (WTD, 21.2% crude fat, E15721-34 from Sniff Spezialdiäten GmbH) for approximately 22 weeks. Age-matched C57BL/6 mice (female, 24-28 g weight, from Charles River Laboratories) were used as controls. Control mice were fed a normal chow diet. Experiments were approved by the local animal care committee (animal license ROB-55.2-2532.Vet_02-17-29.) and were in accordance with the German Animal Welfare Act.

### Production of the probes for Gal3 targeting

Gal3-F(ab')_2_ mAb was produced by pepsin digestion of a well-characterized rat anti-Gal3-mAb (clone M3/38, Mabtech) binding to an amino-terminal common epitope of human and mouse Gal3 21. The digestion was performed after preconditioning of the antibody at acidic pH, as reported earlier 22.

The conjugation with the AlexaFluor488 fluorescent dye and with a Cy5.5-NHS ester derivative, as well as the conjugation to desferrioxamine (DFO) and radiolabeling with ^89^Z was carried out as previously reported (supplementary material) 20.

### Selective binding to human macrophages* in vitro*

CD14-positive monocytes were isolated from peripheral blood mononuclear cells (PBMCs) obtained from healthy donors using positive selection technology (MACS technology; Miltenyi Biotec). Successive differentiation in M1 and M2 macrophages was achieved *via* incubation in specific media and in presence of activating substances and cytokines (supplementary material) 23. Differentiated cells were incubated with 100 nM of AlexaFluor488-Gal3-F(ab')_2_ mAb for 1 h at RT. After incubation, media were removed, cells were washed twice with PBS and imaged under a Biorevo BZ-9000 fluorescence microscope (KEYENCE) using a Plan Fluor (Kodak) 20× lens. Another set of cells was incubated with 10 nM of ^89^Zr-DFO-Gal3-F(ab')_2_ mAb for 1 h at RT, or co-incubated with 100-fold excess of unlabelled full-length rat anti Gal3 mAb (clone M3/38, Mabtech, Nacka, Sweden), referred as blocker (supplementary material). After incubation, media were removed, the cells were washed twice with PBS, detached using Macrophage Detachment Solution DXF (C-41330, PromoCell) and transferred into appropriate tubes to measure the associated radioactivity in an automated gamma counter (PerkinElmer 2480 WIZARD^2^). Data are shown as percentages of cell-associated radioactivity over total radioactivity added. The experiments were performed in triplicate.

### *In vivo* imaging PET/CT

Two groups of mice, ApoE-KO (*n =* 12) and control (*n =* 6), were used for *in vivo* and *ex vivo* imaging. Mice were kept sedated with 1.5-2% isoflurane during injections and PET/CT scans. Static images were acquired 48 h after i.v. injections of ^89^Zr-DFO-Gal3-F(ab')2 mAb (2.2 ± 0.2 MBq, 8-9 µg) using the Inveon small animal PET/CT scanner (Siemens, Knoxville, TN, USA) with an acquisition time of 20 min. Images were reconstructed using Siemens Inveon software, which employs a 3-dimensional ordered subsets expectation maximum (OSEM3D) algorithm without attenuation and scatter correction. To confirm tracer uptake specificity, a group of ApoE-KO mice (*n =* 3) was co-injected with a 100-fold excess of unlabeled full-length rat anti Gal3 mAb, and referred as *ApoE-KO blocked*.

### Biodistribution and *ex vivo* imaging (PET/CT, autoradiography, and Sudan-IV staining)

Tracer biodistribution and organ accumulation analyses were evaluated as previously described 20. Whole length aortas (from the sinotubular junction to the iliac bifurcation) were excised under a dissection microscope (Zeiss Stemi DV4 SPOT) as described earlier 23. Five aortas excised at necropsy from ApoE-KO mice were also scanned *ex vivo* with PET/CT to quantify signal intensity in thoracic and abdominal aorta plaques. Additionally, explanted aortas (*n =* 4 non-blocked, *n =* 2 blocked, and *n =* 2 control) were dissected longitudinally, stained with Sudan-IV for neutral lipids using a previously published method 24,25. Sudan-IV-stained aortas were then exposed to phosphor imaging plates (FUJI IMAGING PLATE, FUJIFILM), and radioactivity signals were collected for 1 week. The imaging plates were scanned using a phosphor imaging system (Raytest, Straubenhardt, Germany), and the autoradiogram images were analysed for count density. Regions of interest (ROIs) on the whole aorta were used to measure total quantum level units (QL) that were present in that area. Data were used to calculate autoradiographic signal intensity (QL/mm^2^) in whole aorta.

### Fluorescence *in vivo* and *ex vivo* imaging Gal3 expression

Four mice (3 ApoE-KO and 1 control healthy mouse) were sacrificed 48 h after i.v. injection of 100 µg of Cy5.5-Gal3-F(ab')_2_ mAb diluted in 300 µl saline solution, embedded in a mixture of Tissue-Tek and black ink (3.2% v/v) inside a cylinder of 4.5 cm diameter, and placed into the -80 °C freezer overnight. Cryosections were obtained using a Leica CM 3500 cryostat (Leica, Wetzlar, Germany) at distances of 20 μm at -17 °C. A highly sensitive fluorescence imaging system was mounted onto the cryostat and macro tissue sections imaged every 5 slices (*i.e.* 100 μm), as reported previously 24-26. Fluorescence excitation source was a continuous wave (CW) laser diode emitting at 670 nm, while detection was achieved through a 710/10 nm bandpass filter by an iXon electron multiplying charge-coupled device (EMCCD, DV8201-BV, Andor Technology, Belfast, Northern Ireland). Serial sectioning and imaging were fully automated using custom software implemented in LabView (National Instruments, Austin, USA) to control sectioning and trigger image acquisition 27. Post-processing and visualization were conducted with MATLAB (Mathworks, Natick, USA) and Amira (FEI Visualization Sciences Group, Burlington, USA).

Whole body imaging was performed on 2 ApoE-KO mice and one healthy mouse, while from the remaining ApoE-KO mouse slices of interest at 10 μm thickness were obtained with a LEICA CM 1950 cryostat. These slices were processed for H&E staining and morphological analyzed with a Zeiss M2 Axio Imager microscope (Carl Zeiss AG, Oberkochen, Germany), while from neighboring unstained slices fluorescent (Cy5.5 filter set; 650/45 nm bandpass filter for excitation and 685 nm longpass filter followed by 720/60 nm bandpass filter for emission) images were captured.

### Immunofluorescence staining (IFS) and confocal microscopy

To visualize macrophages, atherosclerotic lesions of the aorta from ApoE-KO mice (*n =* 2) were stained for CD68 and Gal3 as described previously and in supplementary material 28,29. Secondary antibodies conjugated with Cy5 and Alexa488 were applied. DAPI was used to stain nuclei. Sections were analysed using an SP8 confocal laser scanning microscope (Leica, Mannheim, Germany). For 3D imaging, z-stacks were prepared at 1 μm-intervals with a scan zoom factor of 2 using 100x objective and processed by LasX software (Leica, Mannheim, Germany). ImageJ was routinely used for image processing. All images were saved as TIF files and exported into Adobe Illustrator CS6 for figure arrangement.

### Immunohistochemistry analyses for Gal3 expression in murine aortas and human samples

Gal3 expression on murine and human atherosclerotic plaque sections was assessed by immunohistochemistry using a purified horseradish peroxidase-conjugated version of the rat mAb to Gal3 (10 µg/mL; clone M3/38, Mabtech, Sweden) used for the production of the F(ab')_2_ 30.

For CD68 and α-SMA staining, mouse anti CD68 (clones PG-M1 and KP-1, Abcam, Cambridge, UK) and mouse anti-α-SMA (Clone 1A4, Dako, Denmark A/S) mAbs were used according to the manufactory instructions at concentrations ranging ~10-30 µg/mL. 4-µm-thick longitudinal cross-sections along the axis of the aorta were prepared from formalin-fixed and paraffin-embedded tissues. For Gal3 immunostaining on human and mouse tissue sections, an antigen retrieval microwave treatment in 0.01 M citrate buffer pH 6.0, at 750 Watt for three cycles (3 minutes each) was applied as previously described 30. For CD68 and α-SMA immunostaining, heat-induced epitope retrieval was achieved in a solution containing 10 mM Tris-buffer, 1 mM EDTA pH 9.

For indirect immunoperoxidase, mAbs were incubated at 4°C for 30-45 min in a moist chamber. After washing in PBS the sections were incubated with a secondary goat anti-mouse IgG antiserum-HRP-conjugated (Dako) at dilution 1:200 for 30 min. The enzymatic activity was visualized using 3,3'-diamino-benzidine (Dako, Denmark) as the chromogen.

In order to compare expression of Gal3 in the ApoE-KO model with that in human plaques, 42 surgical samples of human plaques obtained from arteries during surgery (19 women and 23 men) or post-mortem tissues (5 cases), Gal3, CD68 or α-SMA staining were used as described for murine aortas. Images were recorded using an Aperio CS2 ScanScope image capture device (Leica Microsystems, Wetzlar, Germany) and analyzed using Aperio Slide Viewing software (Aperio technologies, CA, USA). Morphological analyses and immunohistochemistry were performed in blind and independently by two experienced pathologists. The study on surgical human samples was carried out according to the ethical guidelines of the Declaration of Helsinki. Specific approval was also obtained from the institutional scientific board and ethical committee of Sant'Andrea Hospital (Prot. CE nr. 8391/2013).

### Single cell RNA sequencing (scRNA-Seq) of murine plaque macrophages

Plaques were dissected from aortas of aged (> 78 weeks) ApoE-KO mice (*n* = 3). Plaques were removed using a dissection microscope and cut into small pieces, then digested with enzyme cocktail (400 U/mL collagenase type I, 10 U/mL collagenase type XI, 60 U/mL hyaluronidase and 60 U/mL DNase I, 20 mM HEPES in DPBS) for 40 min at 37°C with slow shaking. Single cell suspensions were stained with fixable viability dye and the panleukocyte marker antibody CD45 (Ebioscience). Total single live CD45^+^ cells were sorted by FACSAriaTM II. The RNA library of total CD45-positive (CD45^+^) live cells was constructed using the Chromium Single Cell 5' Library & Gel Bead Kit (10X Genomics) and sequencing was performed by 26+ 91 bp paired-end next generation sequencing (Illumina). The sequencing raw data was demultiplexed with the cellranger mkfastq pipeline, reads were aligned to mouse mm10 reference transcriptome using STAR alignment built in Cell Ranger (version 3.1.0). All 578 macrophages from total plaque CD45^+^ cells were sub-clustered for 2000 highly expressed genes of each cell using non-linear dimensional reduction (tSNE) algorithm. Three macrophage subtypes were designated following a previous report using marker gene profiles 31. LGALS3/Gal3 gene expression of each macrophage in murine plaques was shown as violent plots.

### scRNA-Seq of human atherosclerotic plaque macrophages

Human plaque single cell sequencing raw data was downloaded from a publicly available databank (https://figshare.com/s/c00d88b1b25ef0c5c788; doi: 10.6084/m9.figshare.9206387) 31. All (1698) macrophages were sub-clustered into three subpopulations by tSNE 32. Three macrophage populations were defined according to a previous report 31. LGALS3/Gal3 gene expression of each macrophage subtype in human plaques is shown as violent plots.

### Statistics

Data are expressed as means ± standard deviation (SD). The Mann-Whitney U test was used to compare two variables. A *p*-value ≤ 0.05 was considered significant. One-way ANOVA was used to compare results from gene sequencing analysis of three independent groups. Statistical analyses were performed using SPSS Statistics software (version 24.0.0, IBM).

## Results

### Preparation of AlexaFluor488-, Cy5.5-conjugated and ^89^Zr-DFO-labelled Gal3-F(ab')_2_ mAb

The AlexaFluor488-Gal3-F(ab')_2_ mAb and Cy5.5-Gal3-F(ab')_2_ mAb conjugates showed a fluorochrome/protein ratio of 5.6 ± 0.1 and 3.6 ± 0.1, respectively. Labelling of DFO-Gal3-F(ab')_2_ mAb with ^89^Zr resulted in a 74 ± 4% radiochemical yield and a radiochemical purity of 98.5 ± 0.5% after purification. The specific activity of the probe was 26.0 ± 2.0 MBq/nmol.

### AlexaFluor488-Gal3-F(ab')_2_ mAb and ^89^Zr-DFO-Gal3-F(ab')_2_ mAb bind to activated human macrophages, mainly M2 (M2a/M2c) subsets *in vitro*

**Figure 1:**
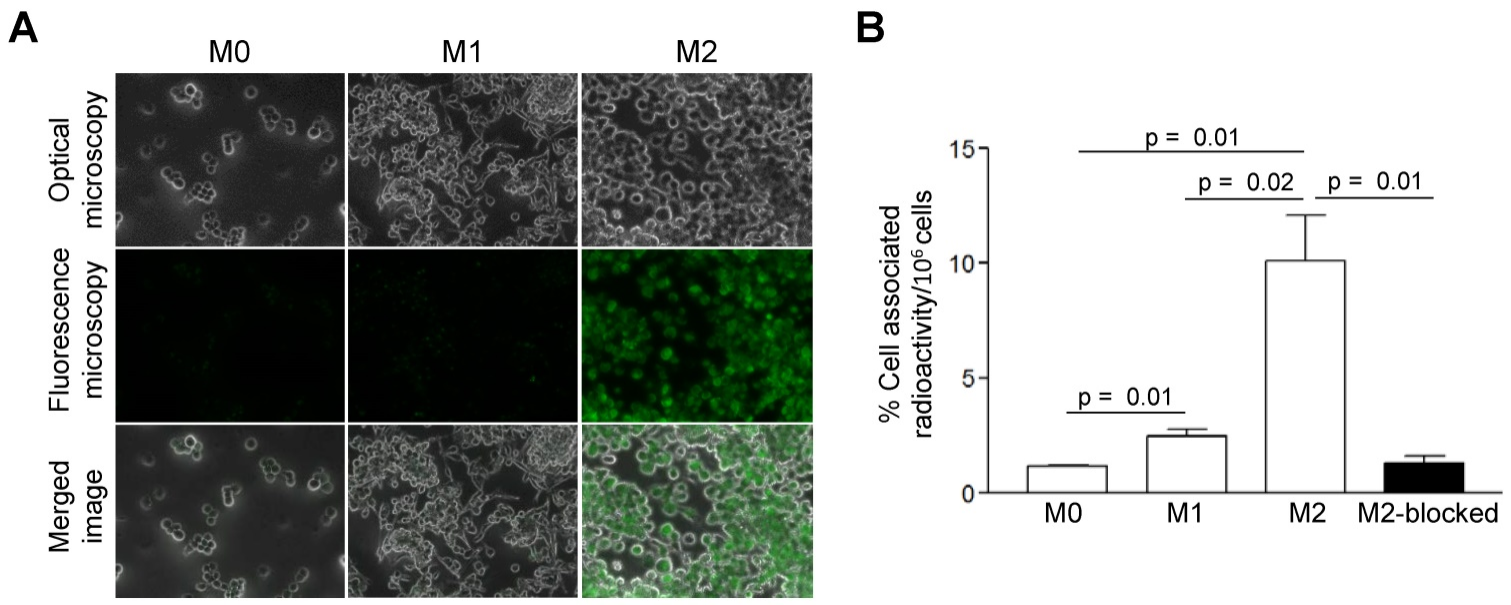
***In vitro* binding-specificity assays. A**,* In vitro* binding selectivity of AlexaFluor488-coupled Gal3-F(ab')_2_ mAb to Gal3 was tested on M0, M1 and M2 (M2a/M2c) polarized macrophages. IL-4/IL-10 activated M2 polarized macrophages displayed significantly enhanced uptake of AlexaFluor488-anti-Gal3-F(ab')_2_ when compared with M0 and M1 macrophages. **B**,* In vitro* binding specificity of ^89^Zr-DFO-Gal3-F(ab')_2_ mAb to Gal3 was tested on M0, M1 and M2 (M2a/M2c) polarized macrophages. M2 (M2a/M2c) macrophages in blocked dishes were co-incubated with a 100-fold excess amount of non-labelled full-length anti Gal3 mAb (mean value of three dishes ± SD). Binding of ^89^Zr-DFO-Gal3-F(ab')_2_ mAb to M2 macrophages was significantly reduced when Gal3 epitopes were saturated with a large molar excess of unlabeled mAb, indicating that binding of ^89^Zr-DFO-Gal3-F(ab')_2_ mAb is specific.

Compared to M0 and M1 macrophages, IL-4/IL-10-activated M2 cells (M2a/M2c) displayed significantly increased uptake of AlexaFluor488-Gal3-F(ab')_2_ mAb (Figure 1A). More than 4- and 8-fold greater cell-associated ^89^Zr-DFO-Gal3-F(ab')_2_ mAb radioactivity was observed for M2 (M2a/M2c) macrophages compared to the M1 and M0 subsets, respectively (Figure 1B). Binding of ^89^Zr-DFO-Gal3-F(ab')_2_ mAb to the M2 (M2a/M2c) macrophages was target-specific, as it could be decreased ~8-fold by co-incubation with a 100-fold excess of non-labelled rat anti-Gal3 mAb (Figure 1B).

### ^89^Zr-DFO-Gal3-F(ab')_2_ mAb accumulates specifically in plaques of ApoE-KO mice

**Figure 2:**
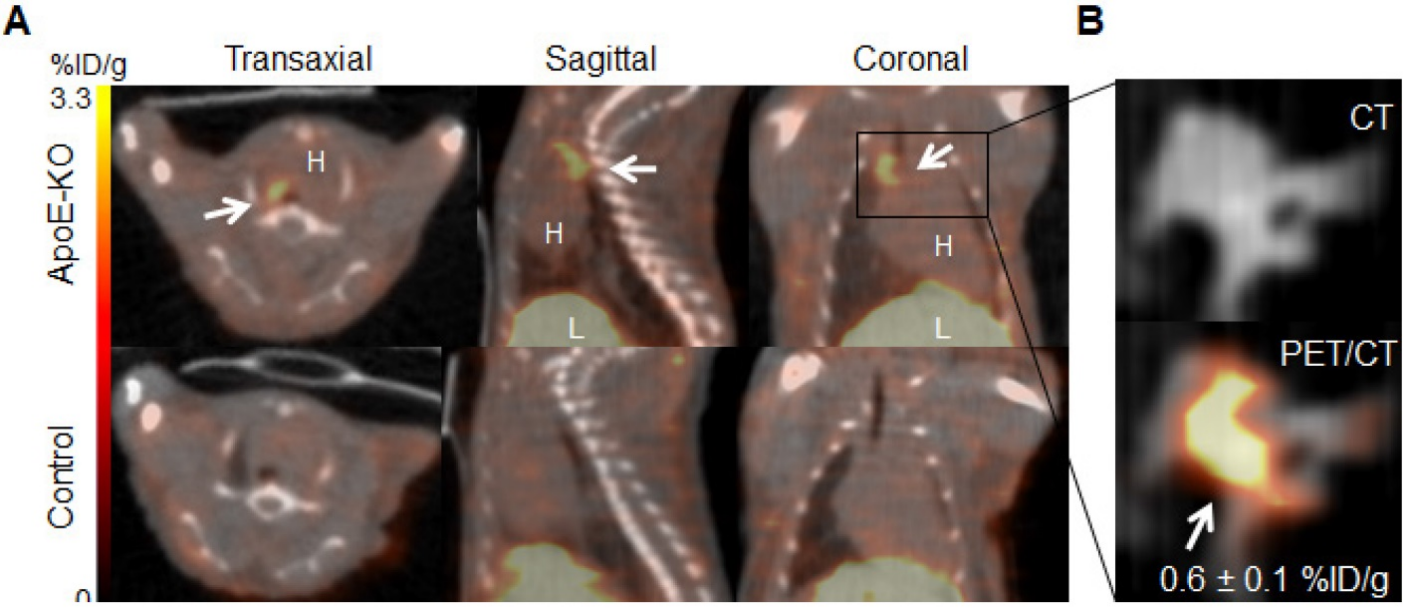
***In vivo* and *ex vivo* PET/CT images. A**,* in vivo* PET/CT images (axial, sagittal and coronal views) of thorax acquired 48 h post injection of ^89^Zr-DFO-Gal3-F(ab')_2_ mAb from ApoE-KO and control mice. Note the intense focal signal in the first tract of thoracic aorta (white arrows). L stands for liver and H stands for heart. **B**, Corresponding *ex vivo* PET/CT image and *ex vivo* PET signal quantification of the plaque in aortic arch. Scale bar accounts for both images.

Representative images of PET/CT scans acquired 48 h p.i. with ^89^Zr-DFO-Gal3-F(ab')_2_ mAb for ApoE-KO and control mice are shown in Figure 2A. The lesions in the aortic arch of ApoE-KO mice are clearly visualized *in vivo*. In contrast, no signals were seen in the aortas of control mice. These findings were confirmed by *ex vivo* PET/CT images that showed ^89^Zr-DFO-Gal3-F(ab')_2_ mAb binding in the same area (Figure 2B). Whole body PET/CT and data of the *ex vivo* distribution of ^89^Zr-DFO-Gal3-F(ab')_2_ mAb in ApoE-KO mice are presented in Online Supplementary Figure 1. Atherosclerotic lesions of the abdominal aorta were only faintly detectable *in vivo* due to physiologic uptake of the Gal3 antibody by the liver, but visualized when analyzed *ex vivo* by PET imaging (Figure S2).

**Figure 3:**
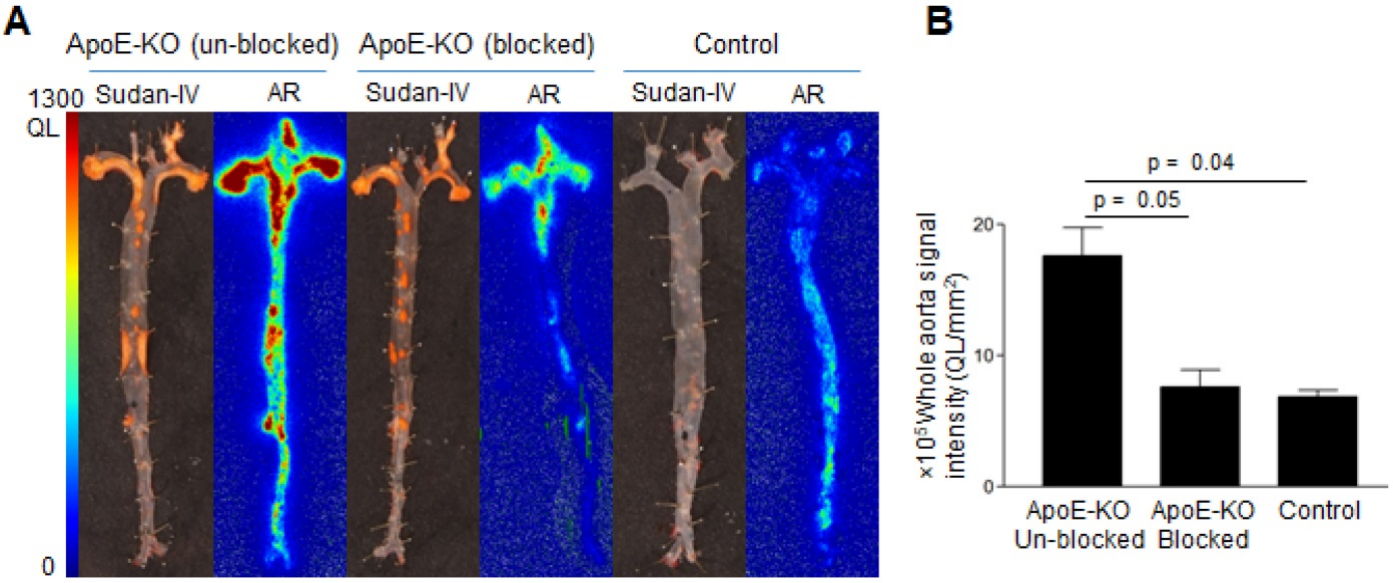
** Sudan-IV staining, autoradiography, and autoradiography signal quantification of ^89^Zr-DFO-Gal3-F(ab')_2_ mAb uptake in mice aorta. A**, Sudan-IV staining and corresponding autoradiography (AR) of aortas excised from ApoE-KO non-blocked, blocked and control mice.** B**, Quantification of the autoradiography images expressed as intensity per unit area of whole aorta (whole aorta autoradiography signal/whole aorta area, QL/mm^2^). Whole aorta areas were quantified using ImageJ software.

Comparative analysis of Sudan-IV staining and autoradiography of aorta segments showed that the radiolabeled antibody accumulated within atherosclerotic lesions (Figure 3A). Aortas from control mice did not exhibit focal radioactivity signal or lipid staining. Quantification of the autoradiography images showed more than 2-fold greater uptake in aortas of ApoE-KO mice ((17.6 ± 3.1) × 10^5^, QL/mm^2^) compared to aortic tissue from control mice ((6.8 ± 0.8) × 10^5^, QL/mm^2^) (Figure 3B). Application of excess unlabeled antibody significantly reduced uptake of radioactivity ((7.6 ± 1.8) × 10^5^ QL/mm^2^, Figure 3B), demonstrating the specificity of Gal3 PET imaging.

### Plaque localization via fluorescent imaging

**Figure 4:**
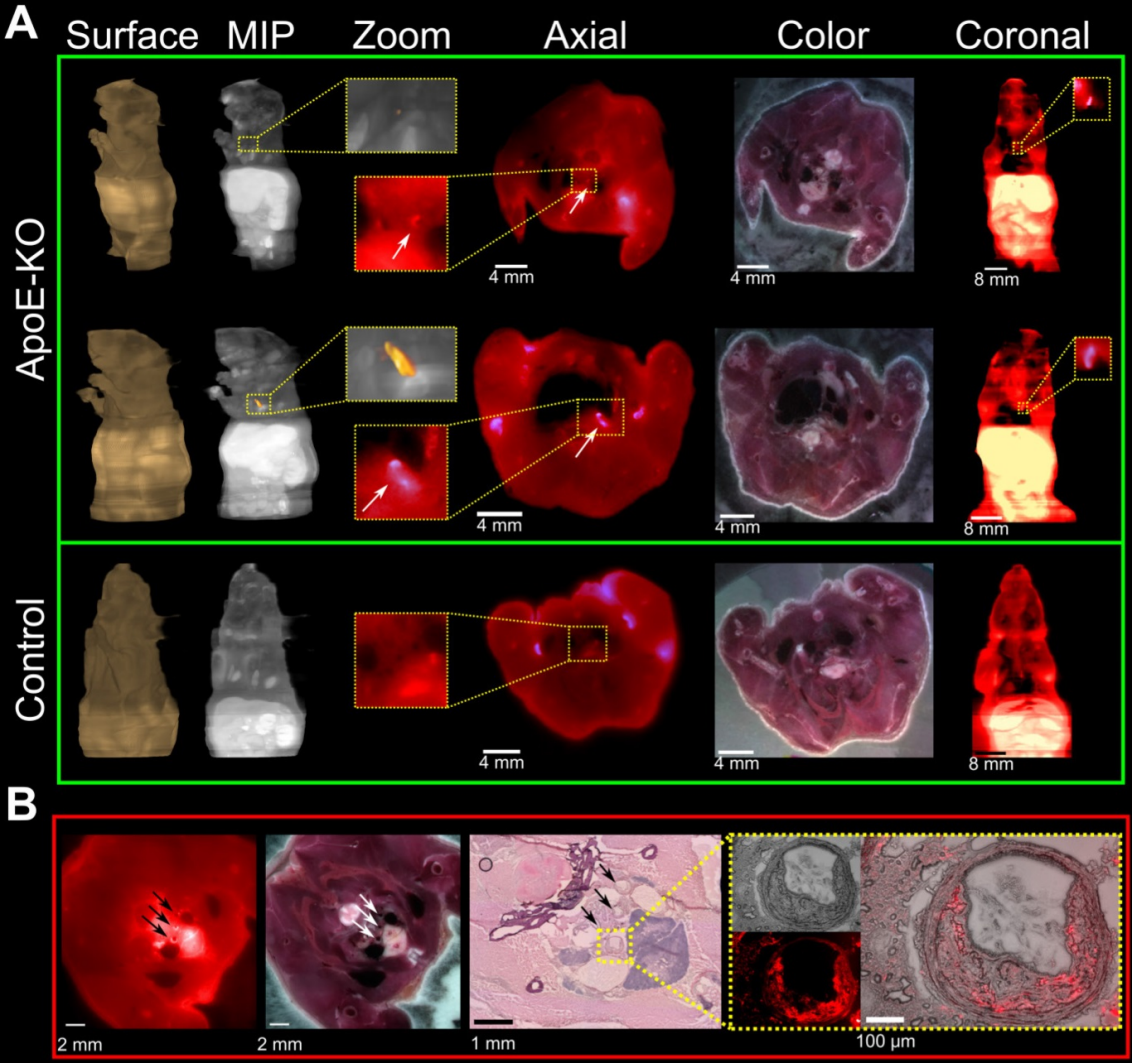
** Fluorescence imaging of plaques on serial mouse sections and histopathology confirmation.** Three ApoE-KO and one healthy mice were injected with 100 µg of Cy5.5-Gal3-F(ab')_2_ mAb and sacrificed 48 h p.i. **A**, ApoE-KO and control mice were sliced and imaged at a step of 100 µm. Atherosclerotic areas could be confirmed in axial and coronal views, despite the relative low contrast due to autofluorescence signal. MIP: maximum intensity projection. **B**, the accumulation of the tracer in the plaques is further confirmed by H&E staining (middle insert figure) and overlapping fluorescence microscopy obtained from one mouse injected with Cy5.5-Gal3-F(ab')_2_. The external arrows indicate sections of epiaortic vessels originating from the aortic arch, while the middle arrow indicates the initial tract of descending aorta that runs alongside the esophagus. The indicated vascular structures show atherosclerotic lesions with different intensity of fluorescent signal (left image). The blue colored structure under the vascular structures represents the thymus. In the yellow box the common arterial trunk is shown with plaque that causes stenosis (H&E top image, right box). Notably, fusion between H&E and florescent images clearly shows accumulation of the tracer in the plaque, likely due to macrophages infiltration (right box). The fluorescence intensities have been normalized to the corresponding maximum values.

Cryosections of mice which were injected with Cy5.5-Gal3-F(ab')_2_ mAb confirmed accumulation of the Gal3 antibody fragment in atherosclerotic lesions (Figure 4A). This was confirmed by comparing the wide-field fluorescence images to conventional morphology at histology and immunofluorescence (Figure 4B and Figure S3). Despite the low contrast due to tissue autofluorescence and the relative limited dimensions of the regions of interest, a prominent fluorescent signal of different intensities distributed inside the plaques was visualized in all vascular structures with atherosclerotic lesions (Figure 4B insert). These findings provide additional evidence for the sensitivity of the presented imaging approach and will guide future studies to confirm the potential of Gal3 intravascular fluorescent imaging.

### Gal3 expression in murine plaques

**Figure 5:**
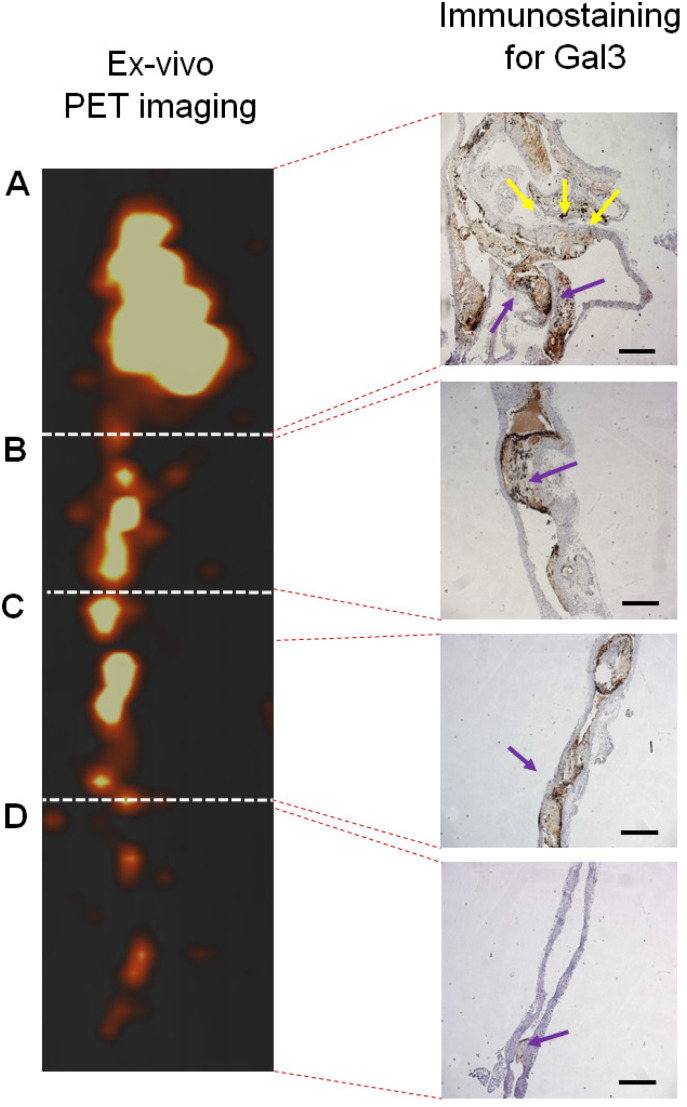
** PET signal versus immunohistochemistry staining for Gal3 of a representative aorta excised from ApoE-KO mouse injected with ^89^Zr-DFO-Gal3-F(ab')_2_ mAb.** Tracer accumulation in different tracts of the aorta was correlating with the brown signal obtained in presence of Gal3 expression in **A**) tract of the origin of aorta with semilunar aortic valve (yellow arrows), which continues with the aortic arch;** B**) tract of thoracic aorta with atherosclerosis lesions (blue arrow); **C**) tract of abdominal aorta with stenotic lesions reach in macrophages (blue arrow); **D**) lumbar aorta presenting slight atherosclerosis (blue arrow). Gal3^+^ macrophages and Gal3 deposition invariably correlate with the signal visualized via PET. Scale bars in IHC images 200 µm. Magnification 10x.

**Figure 6:**
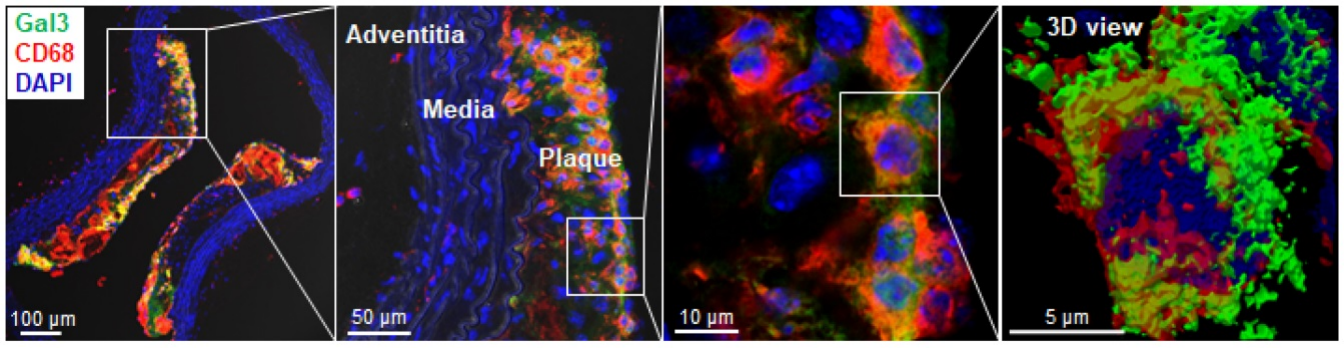
** Localization of Gal3 expressing macrophages in plaques via immunofluorescence staining.** Immunostaining of Gal3 (green), CD68 (red) in 10µm thick ApoE-KO aorta sections. Gal3^+^CD68^+^ macrophages were mainly located in the fibrous cap and shoulder area of the plaque, but not in the media or adventitia (left). Insets showing higher magnifications and 3D reconstructed maximum intensity projection of z-stacks for co-localized expression of Gal3 and CD68 in plaque shoulder area (middle, left). Overlapping domains of expression are shown in yellow. DAPI stained nuclei are shown in blue.

**Figure 7:**
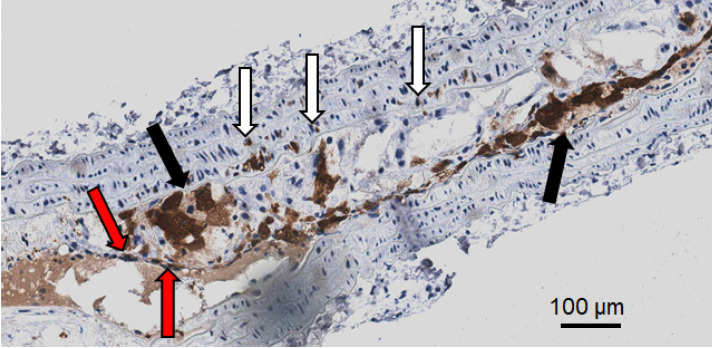
** Representative picture showing different Gal3^+^ cells in a large plaque in 4 µm thick ApoE-KO aorta section.** In dark brown (*black arrows*) Gal3 is visible in foamy macrophages that preferentially express Gal3 in the cytoplasm. Other foamy macrophages are faintly positive or negative at immunohistochemistry in the observed clusters. Scattered smooth muscle cells in the arterial muscular wall also express Gal3 (*white arrows*). Neoformed endothelial cells covering the plaque are also Gal3 positive (red arrows). This picture shows that different cell types in plaques may express Gal3. The proportion of these cell types is variable in each plaque. Magnification 20x.

Immunohistochemical analyses for Gal3 expression performed on murine aortas revealed strong signals in plaques of the aortic arch, and in both the thoracic and abdominal aorta segments (Figure S4). This finding parallels the radioactive signal visualized via autoradiography analyses (Figure 3) and the *ex vivo* PET imaging of excised aortas (Figure S2 and Figure 5). Immunofluorescence staining showed Gal3 expression by CD68-positive (CD68^+^) macrophages and other myeloid cells located in the fibrous cap layer and shoulder region of plaques (Figure 6 and Figure S5). Gal3 expression associated with foamy macrophages as well as scattered smooth muscle cells in the arterial wall was noteworthy. Newly formed endothelial cells covering the plaque were also Gal3 positive (Gal3^+^), indicating ongoing involvement of Gal3 in tissue remodeling (Figure 7). These results show that different cell types in diseased arterial walls may express Gal3 though at different levels of intensity.

### Gal3 expression in human plaques at different stages of disease progression

**Figure 8:**
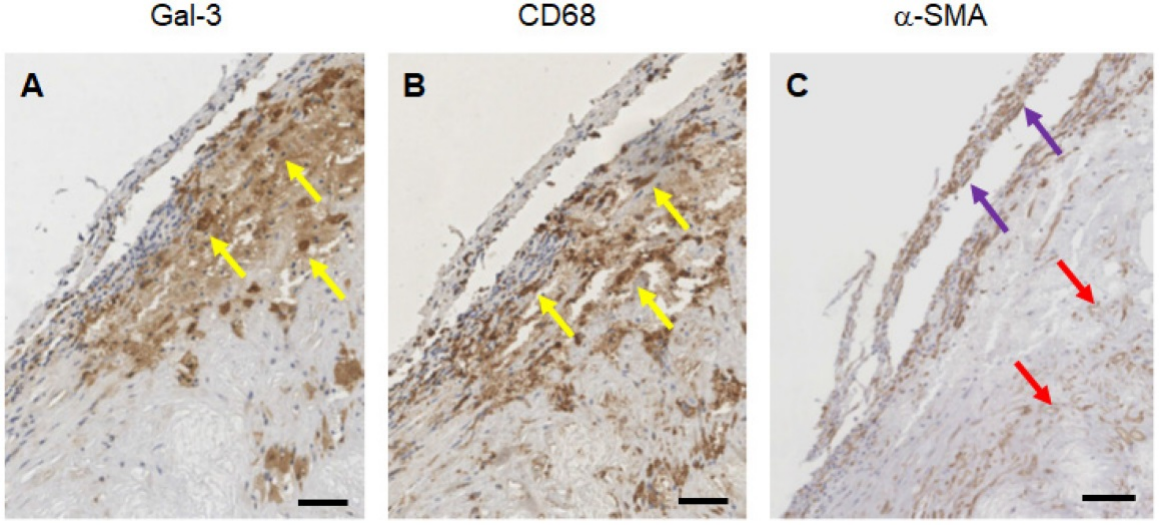
** Immunostaining of human plaques for Gal3, CD68 and α-SMA expression.** A representative panel of immunohistochemical analyses of a large plaque of human aorta is shown. **A**) Gal3 immunostaining using rat mAb to Gal3 clone M3/38 shows positive cells in an atherosclerotic lesion. Some of the cells appear dark-brown, others are faintly stained suggesting different expression level of the lectin protein. **B**) CD68 immunostaining (mAb PG-M1) of the same lesion shows an overlapping signal, which is consistent with a macrophage phenotype (yellow arrows). **C**) α-SMA staining indicate smooth spindle cells (myofibroblast), microvessels and scattered macrophages (see the foamy cytoplasm of some positive cells at the base of the picture, red arrows). Some of these cells express Gal3. Scale bars 200 µm. Magnification 10x.

Expression of Gal3 was detected in all of 47 human samples at different stages of progression. Lesions with signs of vulnerability showed stronger Gal3 signals due to the presence of infiltrates of Gal3^+^ foamy macrophages and activated myofibroblasts (Figure S6). Overlapping expression of Gal3 and CD68 confirmed the presence of Gal3^+^ macrophages in the arterial wall. α-SMA-positive (α-SMA^+^) cells including smooth muscle cells, myofibroblasts (cells which produce extracellular matrix components, primarily collagens), scattered foamy macrophages, and some endothelial cells (which are involved in re-endothelization of the plaque) also showed positive signals for Gal3 (Figure 8). It is noteworthy that Gal3 expression analyses were performed on human plaques which were derived from both male and female subjects and did not show apparent gender-dependent differences.

### LGALS3/Gal3 gene expression on murine and human plaque macrophages *in vivo*

**Figure 9:**
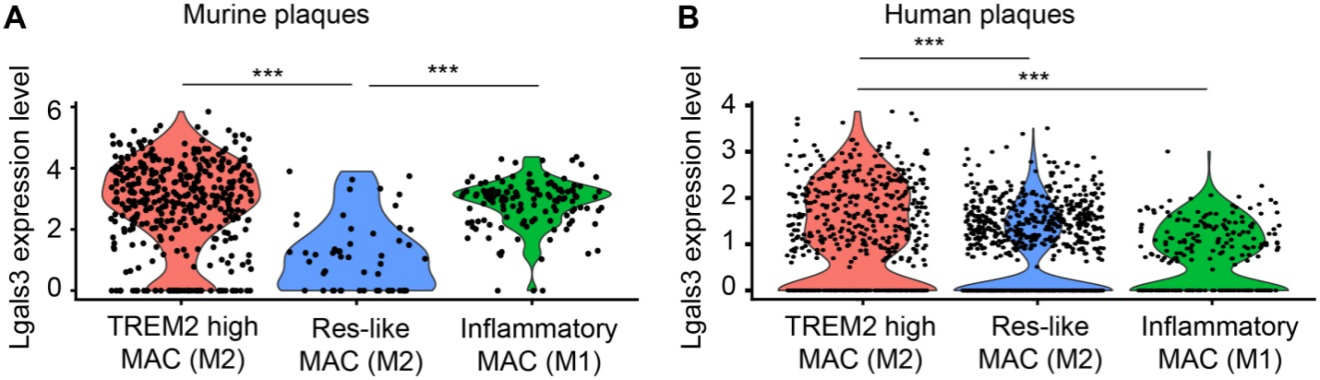
** LGALS3/Gal3 gene expression in plaque macrophages *in vivo.* A**) Single cell sequencing of plaque-derived macrophages of aged ApoE-KO mice revealed LGALS3 gene on three macrophage populations *in vivo*. Each dot represents one individual cell. One-way ANOVA, *n* = 392 TREM2 high macrophages (TREM2 high MAC), *n* = 54 tissue resident-like macrophages (Res-like MAC), *n* = 134 inflammatory macrophages. ***P < 0.001. **B**) Single cell sequencing of human plaque macrophages revealed LGALS3/Gal3 gene on three macrophage populations *in vivo*. Each dot represents one individual cell. One-way ANOVA, *n* = 520 TREM2 high MAC, n = 963 Res-like MAC, n = 215 inflammatory macrophages. ***P < 0.001.

Three different macrophage subtypes were identified in murine plaques: TREM2^high^ macrophages, inflammatory macrophages, and resident-like (Res-like) macrophages. To discriminate TREM2^high^ from resident-like (Res-like) macrophages, the MRC1 (CD11b) gene was utilized as an index marker for M2 macrophages while inflammatory macrophages (M1) were defined by CCR2 gene expression 32. Using scRNA-seq, we analyzed gene expression profiles of 578 plaque macrophages of aged ApoE-KO mice and 1698 macrophages of human plaques (Figure 9). Our data indicated that both M1 and M2 macrophages express Gal3 transcripts. Yet, a portion of TREM2^high^ M2 macrophages express higher levels of Gal3 transcripts when compared to M1 macrophages both in murine and human plaques *in vivo* (Figure 9A, B). These data support the concept that both M1 and M2 macrophages express lgals3/Gal3 and that the number of these transcripts in a subpopulation of M2 macrophages exceed those in M1 macrophages per cell with statistical significance. Further work is required to identify lgals3/Gal3 transcript and protein expression in other myeloid cells including dendritic cells.

## Discussion

Taken together, our findings establish the feasibility of molecular imaging of atherosclerosis by monitoring Gal3 expression on plaques *in vivo* using a PET and fluorescent probe noninvasively. *In vivo* uptake of the antibody fragment was specific for Gal3 expression, as determined by blocking studies. Plaques as small as 3 mm were visualized with high contrast on PET/CT images. At the cellular level, Gal3 immunoreactivity was particularly pronounced in M2 macrophages. Gal3 patterns of expression assessed immunohistochemically in human plaques was similar to the expression pattern evidenced in aortas of the ApoE-KO mice supporting the hypothesis that *in vivo* imaging of the plaques in the murine model can be translated to humans.

Gal3 is a member of the β-galactosyl-binding protein family originally described as cell surface antigen expressed in murine peritoneal macrophages, previously referred to as MAC-2 33. Elevated levels of Gal3 in atherosclerosis have been reported to be associated with macrophages 34. However, the biological role of Gal3 in plaque progression is complex and far from being fully understood 35. In inflammatory tissue environments, Gal3 modulates inflammation and immune responses possibly contributing to activation of immune cells including lymphocytes and macrophages 36, 37. Moreover, Gal3 participates in neoangiogenesis, extracellular matrix remodelling and tissue repair 38,39. Upregulated expression of Gal3 in human atherosclerotic lesions was first reported by Nachtigal *et al.* in plaque specimens obtained from autopsies of trauma victims. In those specimens, Gal3 localized predominantly in macrophages and foam cells and its level of expression correlated with plaque burdens 15, 40. Moreover, Papaspyridonos *et al.* reported that human Gal3 expression is marked in unstable regions of the plaques with high cellularity including foamy macrophages, collagen-producing myofibroblasts, activated newly formed endothelial cells and lipid deposits 16. In view of these data it is conceivable that Gal3 plays clinically relevant roles in atherosclerosis.

Our observations support the hypothesis that plaques harbor several Gal3^+^ cell types of the diseased arterial wall with preferential expression in polarized macrophages though other immune cells, as well as SMCs and endothelial cells also seem to be involved. Fibrous and sclerotic plaques, which are more stable and show scarce cellularity, do not appear to express high levels of Gal3. Novak *et al.* have compared intra- and extracellular expression profiles of Gal3 in classically (M1) and alternatively activated macrophages, and found that Gal3 expression was significantly higher in M2 macrophages with respect to the classically activated cells *in vitro* 13. Our *in vitro* binding results on macrophage subpopulations are consistent with these observations, and show approximately 3-fold higher uptake of ^89^Zr-DFO-Gal3-F(ab')_2_ mAb on M2 (M2a/M2c) macrophages as compared to non-activated and classically activated cells. Moreover, scRNA-seq analyses of plaque-resident macrophages indicate that a portion of TREM2^high^ M2 macrophages express higher level of lgals3/Gal3 transcripts when compared to M1 macrophages both in murine and human plaques. These data indicate that Gal3-F(ab')_2_ mAb-based tracers preferentially bind to M2 macrophages. However, the pathological role of alternative macrophages in atherosclerosis progression and their contribution to plaque (in)stability remains to be fully understood 41-49. Indeed, there is recent evidence for pro-atherogenic properties of alternative macrophages that were previously thought to be anti-atherogenic 44-48. Alternative macrophages may represent important contributors to plaque progression and instability by producing matrix metalloproteinase-9 (MMP-9), which is capable of degrading type IV collagen and triggering plaque rupture 46-49. Yet, ApoE/Gal3 double-KO mice were reported to develop significantly lower numbers of lesions when compared with ApoE-KO mice 50. In a similar study performed by Mackinnon *et al.*, deletion of Gal3 in ApoE-KO mice was associated with smaller plaques with reduced necrotic cores and collagen content 51. More work is needed to clarify the conundrum of pro- versus anti-atherosclerotic impacts of Gal3 in different biological contexts such as age, sex and risk factor burdens 52.

Different experimental imaging approaches, like ultrasound molecular imaging (USI), fluorescent imaging, intravascular photoacoustic imaging, magnetic resonance imaging (MRI) associated to experimental probes have been studied for this purpose 53-56.

Different PET and single photon emission computed tomography (SPECT) radiotracers have been investigated for imaging of plaques 6, 23, 45, 57-59. Among them, Fluorine-18 fluorodeoxyglucose (^18^F-FDG) has become a mainstay of metabolic imaging in atherosclerosis 57, 60. However, the uptake of ^18^F-FDG by macrophages is unspecific and it is taken up by the same membrane transporters that take up glucose in all metabolically active cells. In addition, this approach has limitations in the specific setting of vascular imaging, including physiological uptake in the left ventricular myocardium that impairs imaging of coronary arteries, interaction with blood glucose metabolism, and the need for an extended fasting period before imaging. Due to the high affinity of ^18^F-fluoride to inorganic hydroxylapatite and its ability to localize noninvasively in bone metastasis or soft tissue dystrophic calcification,^ 18^F-sodium fluoride (^18^F-NaF)-PET has also been investigated as a potential noninvasive imaging methodology to identify calcified tissue deposits in plaques with high-risk characteristics 61, 62. By comparing ^18^F-NaF versus ^18^F-FDG in cancer patients, Derlin and co-workers reported contrasting results about accumulation of both tracers in calcified lesions, with colocalization of ^18^F-NaF and ^18^F-FDG occurring in only 6.5% of the arterial lesions studied 63. Considering that not all plaques show calcifications and not all vascular sites of inflammation demonstrate ^18^F-NaF uptake, it has been suggested that fluoride uptake probably occurs in dying foam cells, or in the senescent, dying, foam cells when cell metabolism is reduced or absent 64. These aspects represent intrinsic limitations of this imaging methodology. Experimental probes which bind to receptors expressed on macrophages, like the mannose receptor, have also been investigated 23, 45, 56. The major limitation of these probes lies in the restricted expression of mannose receptors in macrophages. To the best of our knowledge, mannose receptors are not expressed by other cell types involved in plaque remodeling.

The main advantage of using ^89^Zr-DFO-Gal3-F(ab')_2_ mAb over above mentioned tracers is that our probe binds specifically and with high sensitivity to major actors of plaque evolution and vascular repair. This methodology may therefore allow real time imaging of different phases of plaque maturation.

Some limitations associated with the current study require further considerations. First, due to typical late imaging time points and extended patient radiation exposure, the clinical use of an ^89^Zr-labeled F(ab')_2_ fragment for studying plaques could be challenging. To overcome this problem, production of a chimeric/human F(ab') anti-Gal3 mAb with optimized plasma half-life for clinical usage is in progress in our laboratory. Second, the fluorescent dye which was used to label Gal3-F(ab')_2_ mAb was primarily used to study the intra-plaque distribution of the antibody fragment with higher resolution, which is not achievable by autoradiography. Intravascular fluorescence molecular imaging (FMI) is an emerging technology that enables imaging of specific molecular events within rupture-prone plaques 65. Our results support the important possibility that Gal3 expression-imaging may qualify to distinguish stable versus unstable plaques using intravascular FMI, after labeling antibody fragments with near-infrared fluorescent dyes having a longer emission wavelength, such as indocyanine green (ICG) 66.

The role of Gal3 in physiological and pathophysiological processes has led to several distinct galectin inhibitors, both for experimental purposes and potential clinical use as novel therapeutics 67-69. Such inhibitors could be potentially useful to modulate activation of macrophages or other cell types present in plaques. A tracer specific for Gal3 targeting would be of considerable interest to monitor the response to novel therapeutic approaches.

In conclusions, our findings indicate that PET imaging of plaques by targeting Gal3 appears to be feasible. Clinical translation of these imaging approaches holds promise to improve the management of patients affected by plaques at high risk of lethal clinical outcomes of the disease.

## Supplementary Material

Supplementary figures and tables.

## Abbreviations

Gal3: Galectin-3
DFO: desferrioxamine-thioureyl-phenyl-isothiocyanate
^89^Zr: zirconium-89
Fab'_2_: Fragment antigen-binding-2
(ApoE-KO) mice: apolipoprotein E knockout mice
CD68: cluster of differentiation 68 (a monocyte/macrophage marker)
α-SMA: alpha-smooth muscle actin
Cy5.5-NHS: cyanine 5.5 succinimidyl ester
AlexaFluor488-NHS: alexafluor488 succinimidyl ester
PET/CT: positron emission tomography/computer tomography
FMT: fluorescence molecular tomography
H&E: Hematoxylin and eosin staining
HRP: horseradish peroxidase
scRNA-Seq: single cell RNA sequencing
TREM2: Triggering receptor expressed on myeloid cells 2
Res-like macrophages: resident-like macrophages
LGALS3: lectin, galactoside-binding, soluble, 3 gene
^18^F-NaF: ^18^F-sodium fluoride
^18^F-FDG: ^18^F-fluorodeoxyglucose
ICG: indocyanine green
intravascular FMI: intravascular fluorescent molecular imaging
