# Supplementary material for: Imaging atherosclerotic plaques by targeting Galectin-3 and activated macrophages using (89Zr)-DFO- Galectin3-F(ab')2 mAb
Source: Theranostics. 2021 Jan 1;11(4):1864–76. doi: 10.7150/thno.50247 (PMC7778602; doi:10.7150/thno.50247)
Supplement: Supplementary file 1 — Supplementary figures and tables. [file thnov11p1864s1.pdf]

Supplementary material

Methods

Production of the probes for Gal-3 imaging

Anti-Gal3-F(ab')$_2$ mAb was produced by pepsin digestion of a well-characterized rat anti-Gal3-mAb (clone M3/38, Mabtech) having affinity to an amino-terminal common epitope of human and mouse Gal3 (17,18). The digestion was performed after preconditioning of the antibody at acidic pH, as reported earlier (18). The product was purified with a protein G affinity column (Pierce) to remove unreacted mAb and Fc fragments.

The conjugation with the AlexaFluor488 fluorescent dye was performed by adding dropwise a 20-fold excess of AlexaFluor®488-TFP mono reactive ester (GE Healthcare), at a concentration of 10 mg/ml in DMSO, to a solution of 2 mg/ml of Gal3-F(ab')$_2$ mAb (20 µM) in 0.1 M $NaHCO_3$ (pH 9.0). After incubation under stirring at room temperature for 45 min, AlexaFluor488-Gal3-F(ab') mAb was purified by washing in a 30 kDa membrane Amicon Ultra Vivaspin (Merk Millipore) with PBS (pH 7.4). Conjugation of Gal3-F(ab')$_2$ mAb with a Cy5.5 NHS ester derivative was carried out using the above mentioned reaction conditions.

The fluorochrome/protein ratios were determined by P-Class nanophotometer (IMPLEN, Germany), measuring the absorbance of the solution at 280 nm for Gal3-F(ab')$_2$ mAb, and at 494 nm and 680 nm for AlexaFluor®488 and Cy5.5, respectively.

The Gal3-F(ab')$_2$ mAb fragment was conjugated to desferrioxamine (DFO) and labelled with ^{89}Z as described earlier (17).

In vitro binding selectivity and specificity assay on human macrophages

Fresh peripheral blood mononuclear cells (PBMCs) were obtained from healthy donor blood using standard Ficoll-Paque density-gradient (Amersham Biosciences) (19). CD14-positive monocytes were isolated from PBMCs using positive selection technology (MACS technology; Miltenyi Biotec). Isolated cells were plated in 96- and 12-well dishes (10^5 and 10^6cells/dish) respectively, in Monocyte Attachment Medium according to manufacturer's protocol (C28051, PromoCell). Subsequent differentiation was performed by incubating the cells with M0-Macrophage Base Medium DXF (C-28057, PromoCell), M1- or M2-Macrophage Generation Medium DXF (C-28055 or C-28056, PromoCell). M1 activation of macrophages was achieved by addition of IFNγ (50 ng/ml, C-60724, PromoCell) and LPS (100 ng/ml, Sigma-Aldrich) and M2 activation of macrophages was achieved by addition of 20 ng/ml of recombinant human IL-4 and 10 ng/ml of recombinant human IL-10 stimulatory factors (both from Peprotech).

Differentiated cells in 96-well dishes were incubated with 100 nM of AlexaFluor488-Gal3-$F(ab')_2$ mAb for 1 h at room temperature. After incubation, media were removed from dishes and cells were washed twice with PBS and imaged with a Biorevo BZ-9000 fluorescence microscope (KEYENCE) using a Plan Fluor (Kodak) 20x lens.

Cells in 12-well dishes were incubated with 10 nM of ^{89}Zr-DFO-Gal3-$F(ab')_2$ mAb for 1 h at room temperature. One set of M2 dishes was co-incubated with 100-fold excess of non-labelled full-length rat anti Gal3 mAb (clone M3/38, Mabtech, Nacka, Sweden) referred to as M2-blocked. After incubation, media were removed, cells were washed twice with PBS and detached using Macrophage Detachment Solution DXF (C-41330, PromoCell). Cell solutions were transferred into the appropriate tubes. A fraction of cell suspension was used for cell counting. The radioactivity of the remaining cells was measured in an automated gamma

counter (PerkinElmer 2480 WIZARD[2]). The uptake was calculated as percentage of cell-associated radioactivity.

Immunofluorescence staining (IFS) and confocal microscopy

To ascertain the presence of infiltrated macrophages, atherosclerotic lesions of the aorta from ApoE-KO mice (n=2) were immunostained for CD68 and Gal3 as previously reported (24, 25). Briefly, explanted aortas were embedded in Tissue Tec (Sakura Finetek) and frozen on dry ice. 10-µm-thick cross-sections were prepared, thawed at room temperature and fixed for 10 minutes in cold acetone, rehydrated in PBS, blocked with 10% donkey serum, and incubated for 3 hr with primary antibodies diluted with 2.5% bovine serum albumin (BSA). Primary antibodies include rat anti-mouse CD68 (clone FA-11, Bio-Rad) for macrophages and rabbit anti-mouse/rat/human Gal3 antibody (ab53082, abcam). Corresponding secondary antibodies were conjugated with Cy5 and Alexa488. DAPI was used to visualize the nuclei. For negative controls, the staining was performed omitting primary antibodies. Stained sections were analysed using a SP8 confocal laser scanning microscope (Leica, Mannheim, Germany). Fluorophores were visualized by using a 633 nm excitation filter and 650 nm emission long-pass filter for Cy5, and a 488 nm excitation and 505/530 nm emission filter for Alexa488. For 3D imaging, z-stacks were prepared at 1 µm-intervals with a scan zoom factor 2 using 100x objective and then processed by LasX software (Leica, Mannheim, Germany). ImageJ was routinely used for image processing. All images were saved as TIF files and exported into Adobe Illustrator CS6 for figure arrangement.

Supplementary Figures

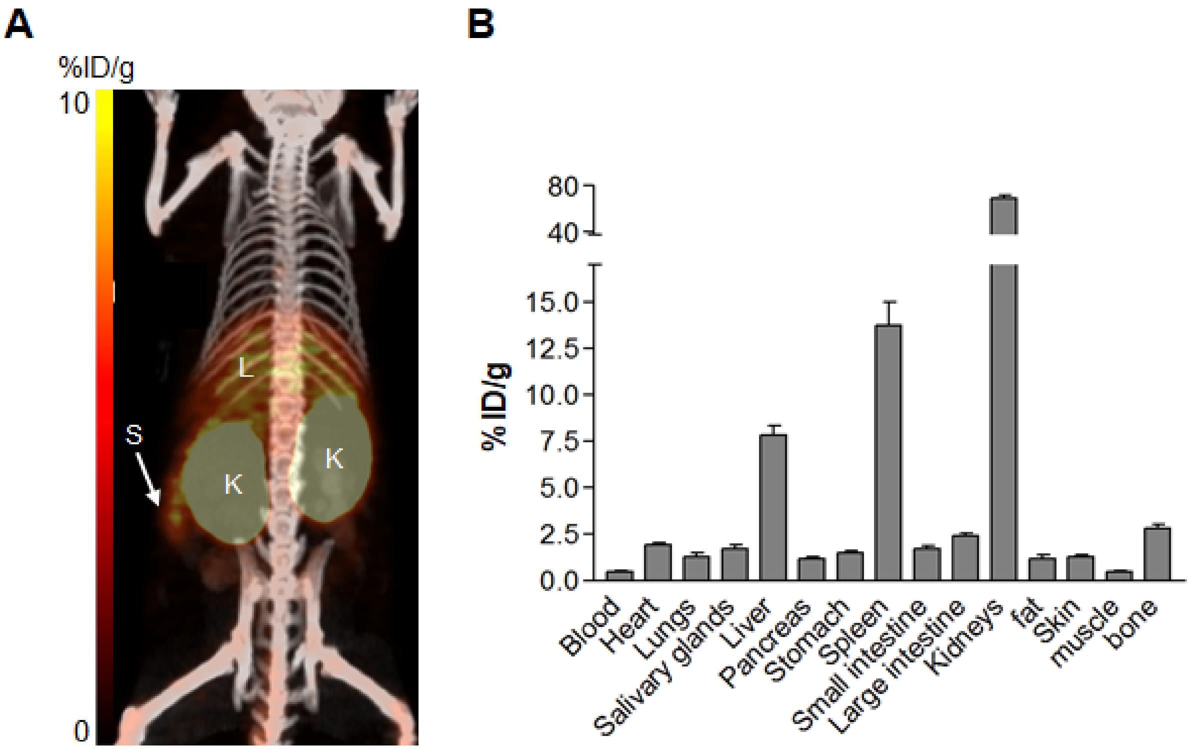

Supplemental Figure 1. A, Whole body *in vivo* PET/CT and **B**, *ex vivo* biodistribution of ^{89}Zr-DFO-Gal3-F(ab')$_2$ mAb in ApoE-KO mice, 48 h p.i. The biodistribution data are presented as the mean percentage of injected dose per gram of tissue (%ID/g ± SD). Data showed the highest accumulation of the radioactivity in the liver (L), spleen (S) and the kidneys (K) due to tracer catabolism.

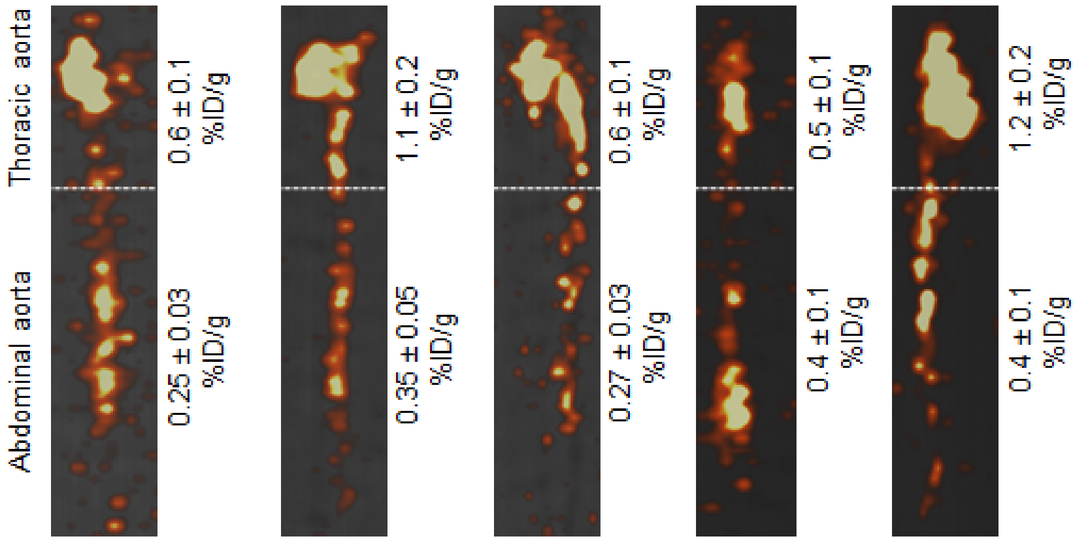

Supplemental Figure 2. Maximum intensity projection (MIP) PET images of five aortas excised from ApoE-KO mice acquired 48 h after injection of ^{89}Zr-DFO-Gal3-F(ab')$_2$ mAb. Tracer accumulation in different tracts of the aorta was measured via image derived uptake calculation using Inveon Workstation Software, and given as mean percentage of injected dose per gram of tissue (%ID/g ± SD).

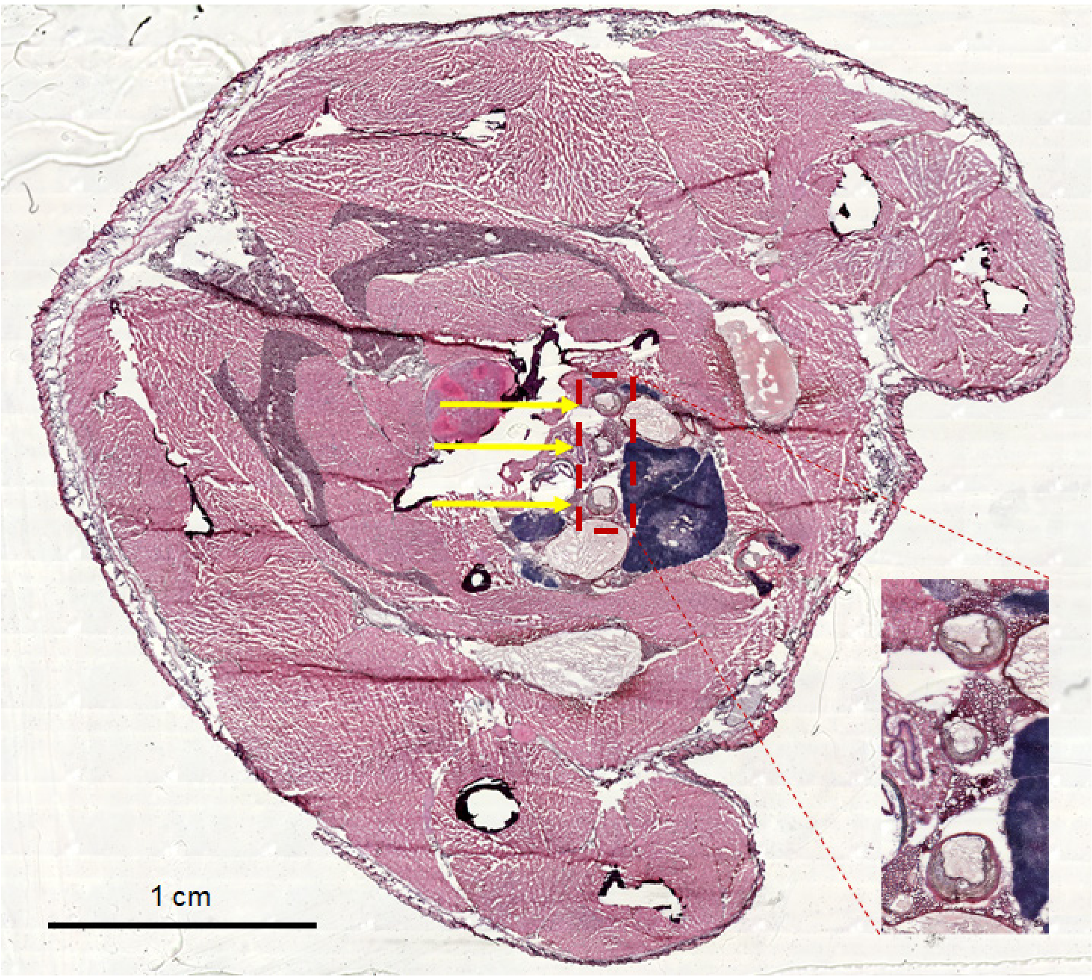

Supplemental Figure 3. Representative H&E immunostaining of an axial section cut in the region over the aortic arch obtained from one ApoE-KO mouse. The mouse was sliced and imaged at a step of 100 µm. The yellow arrows indicate epiaortic vessels originating from the aortic arch (laterals), while the middle arrow indicates the initial tract of descending aorta that runs alongside the esophagous. All vascular structures show obstruction of the lumen due to presence of atherosclerotic plaques (insert).

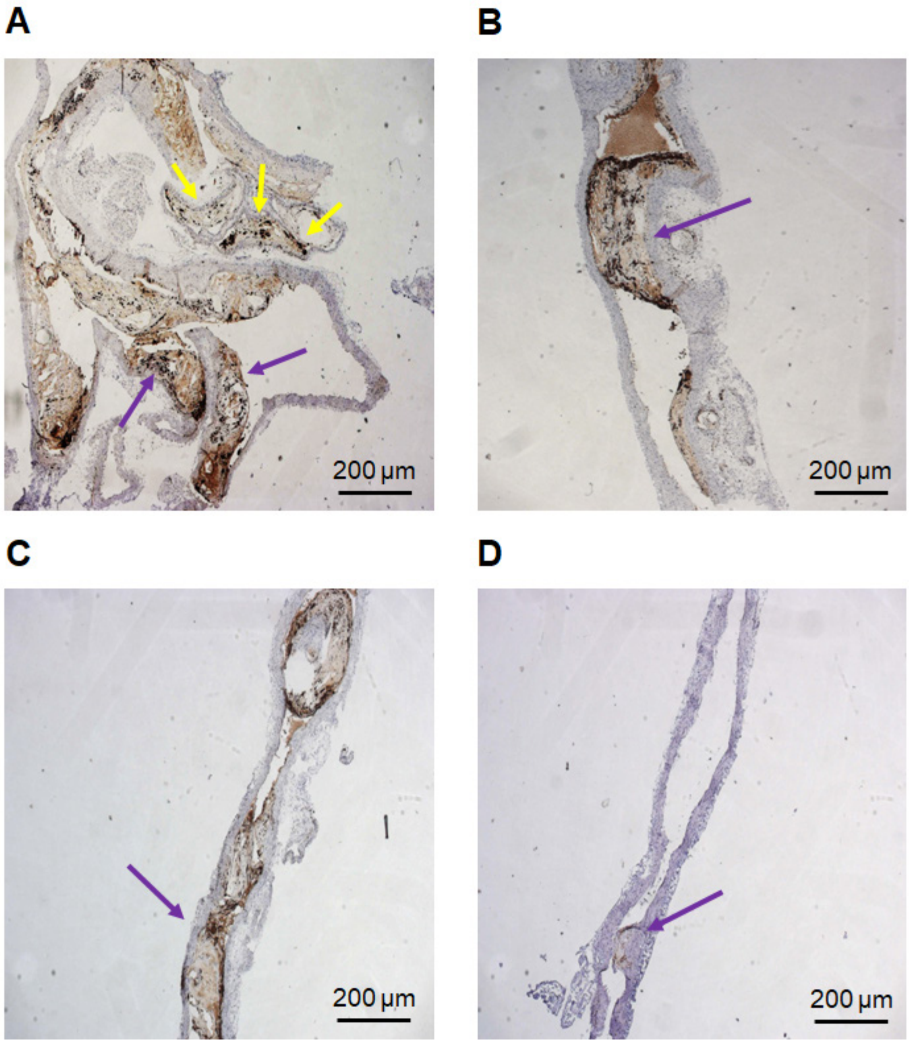

Supplemental Figure 4. Representative images of Gal-3 expression analysis in a whole atherosclerotic aorta explanted from an ApoE-KO mouse.

A) Ascending aorta and aortic arch with semilunar aortic valve (arrows). **B**) Thoracic aorta with atherosclerotic lesions and sub-total lumen stenosis. **C**) Abdominal aorta with different stenotic plaques with macrophages infiltration. **D**) Lumbar aorta with minor atherosclerosis (arrow). $Gal3^{+}$ expression is invariably detected in all of the atherosclerotic lesions. Magnification 10X.

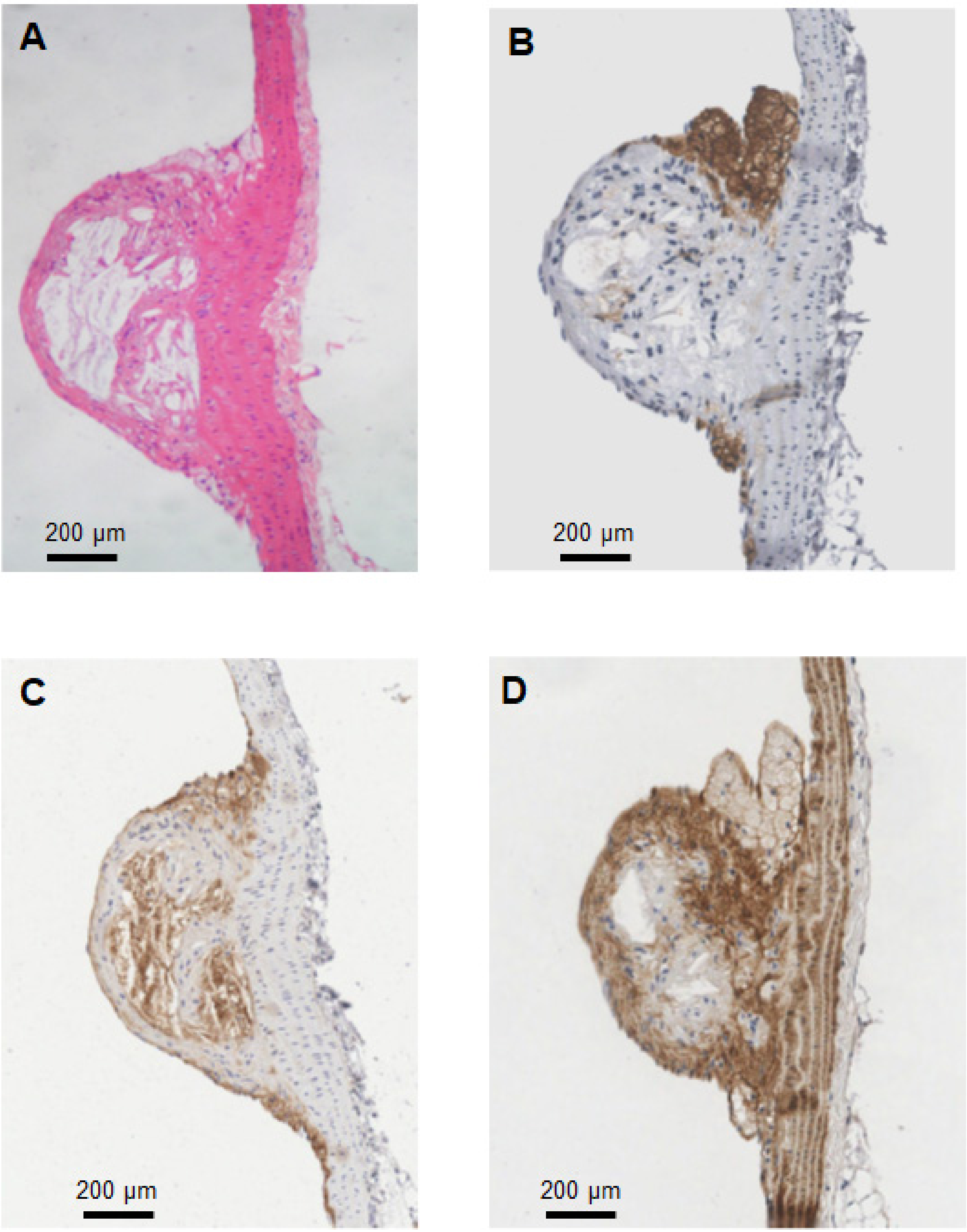

Supplemental Figure 5. Representative figures of tissue sections obtained by longitudinally sectioning a large atherosclerotic aorta from ApoE-KO mouse and stained for Gal-3, CD68 and a-SMA. A) Conventional hematoxylin and eosin (H&E) staining performed for morphological evaluation of atherosclerotic lesions. **B**) Gal-3 immunostaining is shown restricted, in this

specific case, to positive macrophages visible at the periphery of a large atherosclerotic plaque. Scattered foamy cells at the centre of the lesions are faintly stained. C) CD68 immunostaining detects clusters of macrophages at the periphery of the lesion, and depicts aspecifically necrotic areas. A partial overlapping of Galectin-3 and CD68 immunostaining is observed. D) Immunostaining for a-SMA is shown on smooth muscle in the arterial wall and myofibroblasts extended into the lesion. Foamy macrophages in the centre of the lesion as well as at the periphery show a faint unspecific staining. Magnification 10X.

.

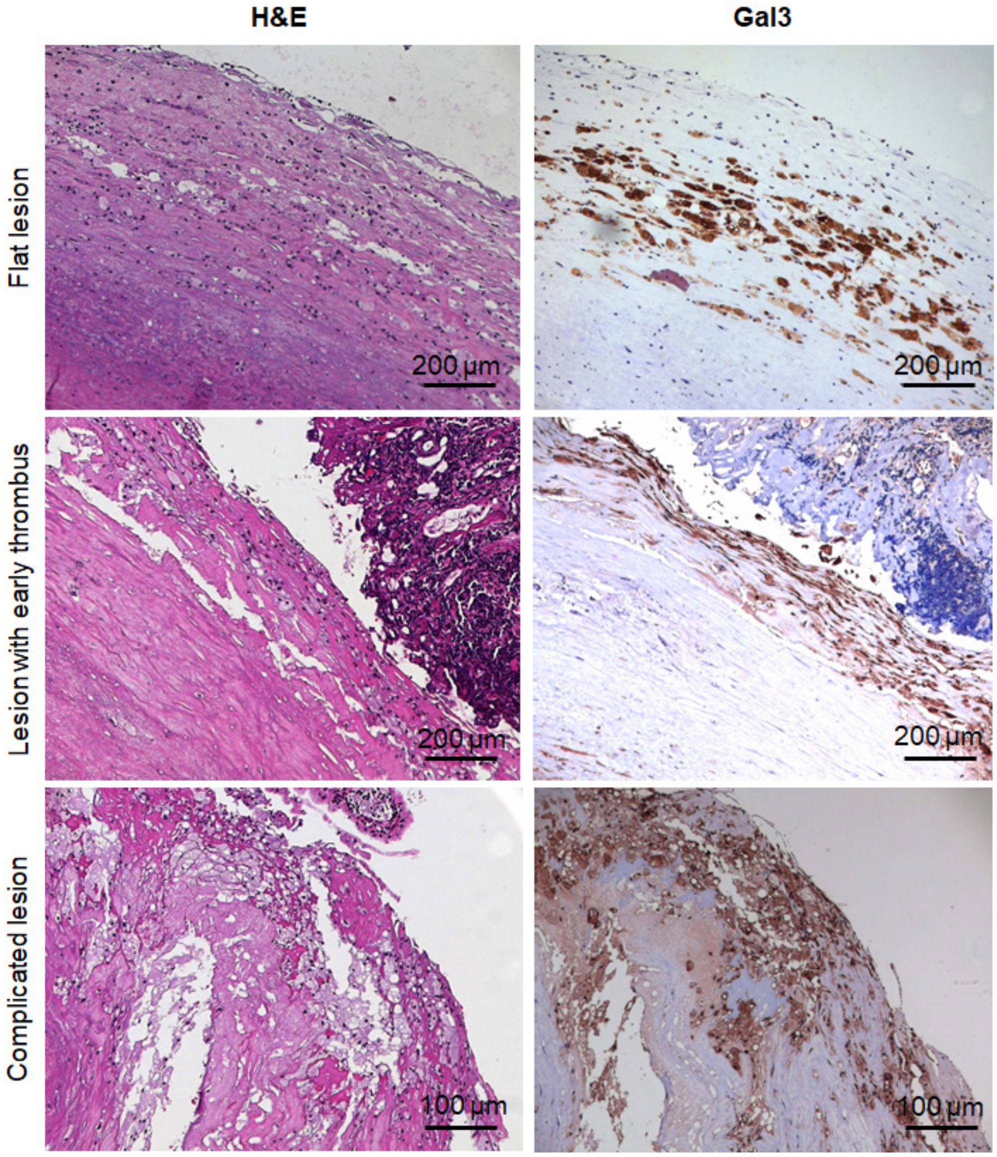

Supplementary Figure 6. Conventional H&E staining and Gal3 expression analysis in human atherosclerotic plaques.

Atherosclerotic plaques from human aorta at different stages of progression are presented. *Upper panel*: In flat lesions (early lesion), infiltrating foamy macrophages expressing Gal3 are visible at sub-endothelial level (tunica intima) in the context of the so called “lipid streak”. *Intermediate panel*: Gal3 staining of an atherosclerotic lesion with disruption of endothelial layer and light remodeling of tunica intima and the superficial portion of tunica media, in which early luminal thrombus is present. The figure shows Gal3^{+} foamy macrophages

commingled with activated spindle vascular smooth-muscle cells (VSMC) of the tunica media, also expressing Gal3. *Lower panel*: IHC staining of a complex atherosclerotic lesion showing a wall dissection infiltrated by massive $Gal3^{+}$ macrophages, with fibrosis and smooth-muscle cells consistently reduced. In this condition rupture of the lesion and severe thrombosis may easily occur. (Direct immunoperoxidase with HRP-conjugated mAb to Gal3). Magnification of figure with slat lesion and early thrombus 20X. Magnification of figure with complicated lesion 10X.
